# Irritable Bowel Syndrome: Contemporary Management Approaches, Limitations, and Future Directions

**DOI:** 10.7759/cureus.110741

**Published:** 2026-06-12

**Authors:** Imran Ahmed, Kunjan Chadha, Keshab R Paudel

**Affiliations:** 1 Internal Medicine, Burrell College of Osteopathic Medicine, Las Cruces, USA; 2 Biomedical Sciences, Burrell College of Osteopathic Medicine, Melbourne, USA

**Keywords:** constipation treatment, diarrhea reduction, irritable bowel syndrome (ibs), low-fodmap diet, treatment of irritable bowel syndrome

## Abstract

Irritable bowel syndrome (IBS) is a common disorder of gut-brain interaction characterized by recurrent abdominal pain associated with altered bowel habits in the absence of structural disease. Despite its high prevalence and substantial impact on quality of life, healthcare utilization, and long-term symptom burden, IBS management remains challenging because of its heterogeneous pathophysiology and variable treatment response. Current evidence supports a multifactorial model involving altered motility, visceral hypersensitivity, dysregulated brain-gut signaling, mucosal immune activation, microbiome changes, and intestinal barrier dysfunction, all of which contribute to the complexity of care. This narrative review summarizes contemporary management approaches for IBS, including dietary interventions, psychological therapies, pharmacologic treatments, biomarker-guided strategies, digital health technologies, and integrated care models. Particular attention is given to the low fermentable oligosaccharide, disaccharide, monosaccharide, and polyol (FODMAP) diet, soluble fiber, cognitive behavioral therapy (CBT), gut-directed hypnotherapy, subtype-specific pharmacologic agents, and emerging digital therapeutics that improve access to behavioral and self-management support. Although the literature demonstrates meaningful benefit across several treatment domains, important limitations remain, including short follow-up duration, lack of mechanistic stratification, inconsistent biomarker validation, and uncertainty regarding long-term treatment sequencing and personalization. Future progress in IBS care will likely depend on precision-oriented approaches that integrate clinical phenotyping, biologic markers, and multimodal treatment pathways tailored to individual patients. Continued emphasis on pragmatic trials, real-world implementation, and mechanism-based care models will be essential to improve outcomes and reduce the overall burden of IBS.

## Introduction and background

Irritable bowel syndrome (IBS) is among the most common disorders of gut-brain interaction and is defined by recurrent abdominal pain associated with altered bowel habits in the absence of structural disease that explains the symptoms [1-3]. Contemporary classification separates IBS into constipation-predominant (IBS-C), diarrhea-predominant (IBS-D), mixed-type (IBS-M), and unclassified forms according to the predominant stool pattern, because symptom phenotype still guides most initial management decisions [1,2].

IBS remains clinically important because of its high prevalence, chronicity, and effect on daily functioning. Patients commonly report impaired quality of life, food-related anxiety, social restriction, missed work or school, and repeated healthcare encounters despite the absence of overt structural pathology [2,3]. At the same time, the therapeutic landscape has expanded rapidly, with growing evidence for dietary therapy, brain-gut behavioral interventions, subtype-specific pharmacologic treatment, digital therapeutics, and mechanism-based care models [4-8]. This review was rewritten to provide a clearer synthesis of contemporary IBS management, major limitations in the current evidence base, and future directions for individualized care.

Methods

This manuscript was prepared as a narrative review of contemporary IBS management. A structured literature search was performed using PubMed/MEDLINE, Google Scholar, and major gastroenterology guideline sources. Search terms included “irritable bowel syndrome,” “IBS,” “low FODMAP diet,” “cognitive behavioral therapy,” “gut-directed hypnotherapy,” “pharmacologic therapy,” “biomarkers,” and “digital therapeutics.”

**Figure 1:**
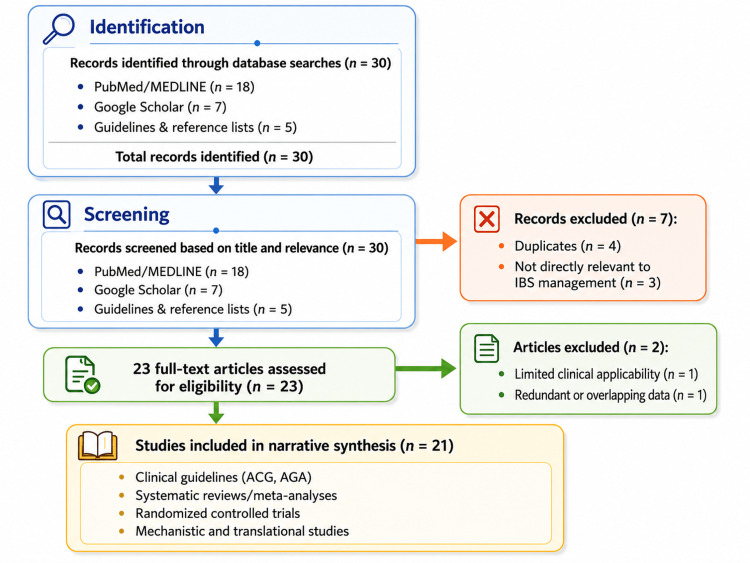
Summary of the literature search and study selection process for this narrative review ACG: American College of Gastroenterology, AGA: American Gastroenterological Association

Records were screened on the basis of title relevance and clinical applicability. Priority was given to clinical guidelines, systematic reviews, meta-analyses, randomized controlled trials, and mechanistic or translational studies published in English. As summarized in Figure 1, 30 records were initially identified, 23 full-text articles were assessed for eligibility, and 21 sources were included in the final narrative synthesis. Because this was a narrative review, the evidence was synthesized qualitatively, and no formal Preferred Reporting Items for Systematic Reviews and Meta-Analyses (PRISMA) workflow or risk-of-bias scoring system was applied.

## Review

Pathophysiology

IBS is best understood as a heterogeneous disorder of gut-brain interaction rather than a single disease entity [2-4]. Multiple mechanisms appear to contribute, including altered gastrointestinal (GI) motility, visceral hypersensitivity, abnormal central pain processing, mucosal immune activation, microbiome shifts, bile acid dysregulation, and impaired epithelial barrier function [2-7]. This broader biologic framework has gradually replaced the older view of IBS as a largely functional or stress-driven diagnosis without measurable pathophysiology.

Immune activation is especially important because it provides a plausible link between dysbiosis, epithelial permeability, and symptom generation. Mast cells and their mediators can sensitize enteric and afferent nerves, contributing to abdominal pain, urgency, and altered motility [5]. At the same time, epithelial barrier dysfunction and microbial changes may promote local inflammatory signaling and abnormal luminal responses in selected patients [4-7]. Mechanistic heterogeneity likely explains why patients with apparently similar symptom patterns often respond differently to the same therapy and supports a move toward more individualized care [6,7].

Dietary interventions

Dietary therapy is commonly used early in IBS management because many patients identify meals as symptom triggers. Food-related symptoms may result from luminal distension, fermentation, osmotic effects, altered motility, and heightened visceral sensitivity [3,8]. Among dietary strategies, the low fermentable oligosaccharide, disaccharide, monosaccharide, and polyol (FODMAP) diet has the strongest evidence base, particularly for bloating, abdominal pain, and global symptom improvement [1,8]. Practical use of this diet should include a structured elimination and reintroduction process under dietitian guidance so that symptom triggers can be identified while minimizing unnecessary long-term restriction [8].

Soluble fiber, especially psyllium, also has consistent benefits and is generally preferred over insoluble fiber such as wheat bran, which may worsen bloating or discomfort in some patients [1,8]. Evidence for a gluten-free diet is less consistent and may partly reflect reduced intake of fermentable carbohydrates rather than a true isolated gluten effect [1,8]. For routine clinical care, broader measures such as regular meals, hydration, moderation of caffeine and alcohol, and avoidance of excess polyols remain reasonable supportive recommendations [8].

Psychological interventions

Psychological interventions have become central to IBS care because symptom severity is shaped not only by bowel habit but also by brain-gut signaling, stress responsiveness, hypervigilance, and avoidance behaviors [2,9-12]. Cognitive behavioral therapy (CBT), gut-directed hypnotherapy, and other brain-gut behavioral therapies consistently improve global IBS symptoms and can reduce abdominal pain intensity [9,10,12]. In practice, CBT for IBS often combines psychoeducation, cognitive restructuring, stress management skills, graded exposure to feared foods or situations, and behavioral strategies aimed at reducing symptom catastrophizing and disability.

Importantly, benefit is not limited to traditional in-person therapy. Minimal-contact and home-based CBT programs have shown meaningful and durable improvement, suggesting that behavioral therapy can be delivered in scalable formats without losing clinical relevance [9-12]. These findings support the idea that brain-gut behavioral therapy should be viewed as a core treatment domain in IBS, not merely as an adjunct reserved for patients with obvious psychiatric comorbidity [10,12].

Pharmacologic treatment

Pharmacologic treatment remains important, especially when symptoms are moderate to severe, subtype-specific, or refractory to initial nonpharmacologic measures. For IBS-D, agents with the strongest evidence include 5-hydroxytryptamine-3 receptor antagonists such as alosetron and ramosetron, rifaximin, and eluxadoline [1,13,14]. These therapies target different mechanisms, including visceral pain signaling, motility, and microbial modulation, but their use must be balanced against adverse effects and patient-specific contraindications.

For IBS-C, secretagogues such as linaclotide, lubiprostone, and plecanatide have demonstrated benefit for constipation, global symptoms, and in some cases abdominal pain [1,14]. Prokinetic therapy may be useful in selected patients with prominent constipation. Across subtypes, neuromodulators such as tricyclic antidepressants and selective serotonin reuptake inhibitors may lessen pain and overall symptom burden, while antispasmodics can offer modest short-term relief [10,14,15]. Overall, medication therapy works best when it is matched to bowel pattern and integrated with dietary and behavioral strategies rather than used in isolation.

Biomarker-guided approaches

Biomarker-guided care is attractive because it could improve diagnostic confidence and treatment selection, but truly validated biomarkers for precision IBS management remain limited [6,7,16]. At present, tests such as fecal calprotectin and fecal lactoferrin are most useful for identifying patients who may have inflammatory bowel disease rather than IBS, thereby helping clinicians avoid diagnostic misclassification [7,16].

Other tools, including colonic transit studies, bile acid malabsorption testing, carbohydrate malabsorption testing, and selected genetic markers, may eventually help define biologically distinct IBS subgroups [6,7,16]. However, the current evidence is not yet strong enough to support routine mechanism-based stratification in most practice settings. Biomarker-informed care is therefore promising, but it remains an emerging rather than fully mature component of IBS management.

Digital health technologies

Digital health technologies have expanded quickly and now offer symptom tracking, structured education, self-management tools, and remote delivery of brain-gut behavioral interventions [15,17-20]. Both real-world observational data and early randomized studies suggest that digital therapeutics can reduce symptom severity and improve quality of life in some patients with IBS [15,17-20]. Their main strengths are scalability, convenience, and improved access for patients who have limited availability of trained behavioral therapists or specialized GI care.

That said, digital platforms are not interchangeable. Differences in content quality, adherence support, therapist involvement, cost, and outcome measurement make it difficult to compare products directly [17-20]. More pragmatic comparative studies are needed before digital therapeutics can be sequenced confidently alongside standard office-based care.

Integrated care models

Given the multifactorial nature of IBS, contemporary management increasingly favors integrated care models that combine dietary modification, behavioral therapy, pharmacologic treatment, and symptom monitoring within a single longitudinal framework [6,17,21]. This approach better reflects clinical reality, where symptom burden fluctuates over time and patients often require more than one therapeutic modality.

Integrated care also allows treatment to be adjusted according to symptom phenotype, patient preference, comorbid anxiety or depression, response to prior therapy, and access to subspecialty resources. Digital tools may further strengthen this model by extending behavioral care, tracking symptoms between visits, and reinforcing dietary adherence [15,17-20]. Although formal integrated IBS pathways still need better long-term evaluation, they represent a practical direction for modern management.

Critical appraisal of the evidence

The contemporary IBS literature has several notable strengths. Multiple randomized controlled trials, systematic reviews, and network meta-analyses now support the use of dietary interventions, psychological therapy, and subtype-specific pharmacologic agents [8-10,13,14]. Many studies also use patient-centered outcomes such as global symptom response, abdominal pain, and quality of life, which improves the clinical relevance of the evidence.

Important limitations remain, however. Many studies are short in duration, frequently measuring outcomes over only several weeks to a few months, which leaves uncertainty about long-term durability, relapse, maintenance therapy, and adherence [17-20]. Mechanistic stratification is also limited, so most trials still enroll broad Rome-defined IBS populations rather than biologically characterized subgroups [6,7,16]. In addition, dietary and psychological studies are difficult to blind, while pharmacologic trials may have narrower eligibility criteria that limit real-world generalizability [8-10,13,14]. These issues make it clear that future progress will depend on longer pragmatic studies and better methods for matching patients to mechanism-based therapy.

## Conclusions

IBS is a heterogeneous chronic disorder of gut-brain interaction that requires more than simple symptom suppression. Current evidence supports a multimodal treatment strategy built on dietary therapy, brain-gut behavioral interventions, subtype-directed pharmacologic treatment, and selected digital tools, with treatment choices tailored to symptom pattern, patient goals, and resource availability.

Even with meaningful progress, important gaps remain in biomarker validation, long-term comparative effectiveness, and treatment sequencing. Future advances in IBS care will likely come from precision-oriented approaches that integrate clinical phenotype, biologic signals, and pragmatic multidisciplinary care pathways. Until then, the most effective care remains individualized, longitudinal, and flexible enough to adapt to the changing needs of each patient.

## References

[1] Camilleri M (2021). Diagnosis and treatment of irritable bowel syndrome: a review. JAMA.

[2] Ford AC, Sperber AD, Corsetti M (2020). Irritable bowel syndrome. Lancet.

[3] Ford AC, Lacy BE, Talley NJ (2017). Irritable bowel syndrome. N Engl J Med.

[4] Aggeletopoulou I, Papantoniou K, Pastras P, Triantos C (2025). Unraveling the pathophysiology of irritable bowel syndrome: mechanisms and insights. Int J Mol Sci.

[5] Aguilera-Lizarraga J, Hussein H, Boeckxstaens GE (2022). Immune activation in irritable bowel syndrome: what is the evidence?. Nat Rev Immunol.

[6] Camilleri M, Boeckxstaens G (2023). Irritable bowel syndrome: treatment based on pathophysiology and biomarkers. Gut.

[7] Shin A, Staller K, Levinthal DJ (2025). Actionable clinical features and biomarkers to facilitate the management of irritable bowel syndrome. Am J Gastroenterol.

[8] Chey WD, Hashash JG, Manning L, Chang L (2022). AGA clinical practice update on the role of diet in irritable bowel syndrome: expert review. Gastroenterology.

[9] Black CJ, Thakur ER, Houghton LA, Quigley EM, Moayyedi P, Ford AC (2020). Efficacy of psychological therapies for irritable bowel syndrome: systematic review and network meta-analysis. Gut.

[10] Ford AC, Lacy BE, Harris LA, Quigley EM, Moayyedi P (2019). Effect of antidepressants and psychological therapies in irritable bowel syndrome: an updated systematic review and meta-analysis. Am J Gastroenterol.

[11] Lackner JM, Jaccard J, Krasner SS, Katz LA, Gudleski GD, Holroyd K (2008). Self-administered cognitive behavior therapy for moderate to severe irritable bowel syndrome: clinical efficacy, tolerability, feasibility. Clin Gastroenterol Hepatol.

[12] Goodoory VC, Khasawneh M, Thakur ER (2024). Effect of brain-gut behavioral treatments on abdominal pain in irritable bowel syndrome: systematic review and network meta-analysis. Gastroenterology.

[13] Black CJ, Burr NE, Camilleri M (2020). Efficacy of pharmacological therapies in patients with IBS with diarrhoea or mixed stool pattern: systematic review and network meta-analysis. Gut.

[14] Lacy BE, Pimentel M, Brenner DM, Chey WD, Keefer LA, Long MD, Moshiree B (2021). ACG clinical guideline: management of irritable bowel syndrome. Am J Gastroenterol.

[15] Pathipati MP, Scott LL, Griser AC, Staller K (2024). Real-world outcomes for a digital prescription mobile application for adults with irritable bowel syndrome. Neurogastroenterol Motil.

[16] Brenner DM, Ladewski AM, Kinsinger SW (2024). Development and current state of digital therapeutics for irritable bowel syndrome. Clin Gastroenterol Hepatol.

[17] Tayama J, Hamaguchi T, Koizumi K (2024). Efficacy of an eHealth self-management program in reducing irritable bowel syndrome symptom severity: a randomized controlled trial. Sci Rep.

[18] Hunt M, Miguez S, Dukas B, Onwude O, White S (2021). Efficacy of Zemedy, a mobile digital therapeutic for the self-management of irritable bowel syndrome: crossover randomized controlled trial. JMIR Mhealth Uhealth.

[19] Eisele M, Yousuf M, Haskey N, D'Silva A, Nasser Y, Franco L, Raman M (2025). Smartphone application with health coaching facilitates multi-symptom improvement in IBS patients: a pilot feasibility trial. Neurogastroenterol Motil.

[20] Shah ED, Salwen-Deremer JK, Gibson PR, Muir JG, Eswaran S, Chey WD (2022). Comparing costs and outcomes of treatments for irritable bowel syndrome with diarrhea: cost-benefit analysis. Clin Gastroenterol Hepatol.

[21] Aggeletopoulou I, Karaivazoglou K, Kalafateli M, Triantos C (2025). Targeting irritable bowel syndrome through diet and mechanism-based therapies: a pathophysiological approach. Nutrients.

